# The socioeconomic impacts of Rift Valley fever: A rapid review

**DOI:** 10.1371/journal.pntd.0012347

**Published:** 2024-08-29

**Authors:** Luke O’Neill, Simon Gubbins, Christian Reynolds, Georgina Limon, Kyriaki Giorgakoudi

**Affiliations:** 1 HSRM Department, School of Health and Psychological Sciences, City, University of London, London, United Kingdom; 2 The Pirbright Institute, Pirbright, United Kingdom; Hokkaido University Research Center for Zoonosis Control, JAPAN

## Abstract

Rift Valley fever (RVF) is a neglected vector-borne disease which is endemic in many countries across Africa and has seen recent geographical expansions into the Arabian Peninsula. RVF can cause severe infections in both animals and humans. RVF infections in livestock can lead to mass fatalities. In humans, the symptoms are nonspecific and can often lead to misdiagnosis. However, a small proportion progresses to haemorrhagic infection with a significantly higher mortality rate. The culmination of this can cause severe socioeconomic impacts.

This review aims to identify the main socioeconomic impacts caused by RVF outbreaks as well as existing knowledge gaps.

Ninety-three academic and grey papers were selected, covering 19 countries and 10 methodological approaches. A variety of socioeconomic impacts were found across all levels of society: Livestock trade disruptions consequently impacted local food security, local and national economies. Most livestock farmers in endemic countries are subsistence farmers and so rely on their livestock for sustenance and income. RVF outbreaks resulted in a variety of socioeconomic impacts, e.g., the inability to pay for school fees. Main barriers to vaccine uptake in communities were lack of access, funds, interest along with other social aspects. The occupational risks for women (and pregnant women) are largely unknown.

To our knowledge, this is the first review on RVF to highlight the clear knowledge gap surrounding the potential gender differences on risks of RVF exposure, as well as differences on occupational health risk in pastoral communities. Further work is required to fill the gaps identified in this review and inform control policies.

## Author summary

Rift Valley fever (RVF) outbreaks have affected many regions globally with varying severity. The number of reported cases in animals and humans is patchy at best, with some countries not recording animal events. This review focuses on 3 main themes: Health Burden; Economic Impacts; and Prevention and Surveillance. The review explores socioeconomic impacts across local, regional, and national levels. We found women have varying degrees of risk to exposure of RVF; however, more research is required to better understand gender differences. RVF outbreaks tend to begin in livestock and spillover into humans. The complex disease dynamics are poorly understood because of the interactions between animals, humans, and the environment. This cross-boundary dynamics fall outside of the public health remit and into veterinary health. This allows for outbreaks to spread uncontrollably for some time. More research using a One Health Lens is required which explores these complex dynamics with the combination of different social aspects. This review highlights the wider impacts experienced at different levels of society and where there are major gaps in our knowledge. Further analysis into these gaps is required to and will aid policy makers in developing new control measures around RVF.

## 1. Background

Rift Valley fever (RVF) is an important neglected zoonotic disease that poses a major threat to global health security without available countermeasures. This has led the WHO to list RVF as a priority pathogen for research and development for preparedness and response to public health emergencies [1]. RVF is endemic to Africa and has seen a recent geographical expansion to the Arabian Peninsula. RVF can have severe consequences in the food system as RVF impacts the productivity and survival of a wide variety of livestock, including camels, cattle, goats, and sheep [2,3]. A small proportion of RVF infections in human populations can develop into haemorrhagic fever leading to high fatality rates [4–6]. Moreover, a recent study in Sudan has shown vertical transmission to be possible in humans [7]. In summary, RVF has the potential to cause public health emergencies due to RVF’s high epidemic potential in both animals and humans and the lack of effective vaccines or treatment.

The causative agent of RVF, Rift Valley fever virus (RVFV), is transmitted by insect vectors, primarily *Aedes* and *Culex* mosquitoes [3,5,8]. RVF outbreaks tend to occur following heavy persistent rain and flooding events because these are the optimal conditions for mosquito population blooms [5,8,9]. Vertical transmission among vectors is possible and mosquito eggs can lay dormant in the soil for decades and hatch once the conditions are suitable [10]. Range expansion of RVFV vectors due to climate change [2] could lead to future epidemics in new areas. Moreover, during interepizootic periods low-level circulation has been demonstrated in both livestock and humans in high-risk occupations [5,11].

The activities of pastoral communities often compete with environmental conditions for natural resources of pasture and water to support their livestock [12]. There are multiple transmission routes for humans to get infected with RVFV. Humans can be bitten by infected mosquitoes or exposed to RVF infected animal tissues, bodily fluids, and animal products (e.g., un-boiled milk) [2,6,9,13]. The majority of RVF outbreaks tend to occur in rural low-resource areas with frequent contact at the animal-human-vector interface. These areas may be inaccessible due to floods and not have the diagnostic capacity for confirming suspected cases, allowing RVF to transmit uncontrollably until confirmation is received. Therefore, this lag could mean it is too late to control the outbreak resulting in more fatalities in both animals and humans [12,14,15].

Once an RVF outbreak occurs in susceptible naive populations the disease tends to become endemic. This can be seen through the introduction of RVF in Saudi Arabia in 2000 [4]. The movement of infected livestock, amplification by vectors and favourable conditions can lead to major epidemics and mass fatalities in susceptible livestock. Livestock species have varying degrees of susceptibility, though the reasons for this host variation are not fully understood [16]. RVFV infection leads to higher rates of abortion and mortality in young ruminants [16]. One potential explanation for this could be due to a lack of previous exposure to RVFV.

Mass fatalities of pastoral herds can lead to food and nutrition insecurity at household level. In addition, the agricultural sector is a major driver of many countries’ economies and societal wellbeing. Increased globalization has enabled increased volumes of international trade. Many RVF endemic countries are highly reliant on exporting livestock. Therefore, RVF outbreaks will cause stakeholders within the food system to react in an attempt to minimise the impact, e.g., trade embargoes, affecting actors across the entire food system.

The aims of this rapid review are to gain a greater understanding of the socioeconomic impacts of RVF and the wider societal factors relating to RVF transmission through data that has been studied and documented in the public domain. The aims of the review were to determine what are the different socioeconomic impacts at different levels of society, i.e., local, regional, and national levels.

## 2. Methods

A search of published studies on PubMed was conducted on the 10 January 2023; all papers returned from the search terms below were considered for inclusion in the review. Standard search terms were developed to collect information on the socioeconomic impacts of RVF.

((Rift Valley Fever OR RVF)

AND

(“Economic Evaluation” OR “Economic Outcomes” OR “Economic” OR “Econ*” OR (“Economic”) OR “Cost-benefit analys*” “Cost-benefit” OR (“Cost”) OR “Financ*” OR (DALYs) OR Disability-adjusted life years) OR QALYs) OR Quality-adjusted life year)

AND

(rift valley fever) OR rift valley fever virus)

AND

(“Socioeconomic” OR “Socioeconomic factors” OR “Socioeconomic variables”)).

The search was conducted for English language studies published in the electronic database PubMed for an unlimited period until January 2023. Broad search terms were purposefully used to increase the probability of capturing all relevant studies.

### 2.1 Screening and data extraction

The data extraction was conducted by one person with co-authors providing advice when necessary.

The first screening stage included reading the title and abstract. The title and abstract had to provide information on one of the following to be included: the economic impact of RVF, wider social impacts of the disease (e.g., socioeconomic impact, epidemiology, environmental), using a variety of methodological approaches (e.g., mathematical modelling, vaccinations, reviews, and risk analysis). In addition, studies which contained outbreak, epidemiological and vaccination data (both quantitative and qualitative), and mathematical models were included. The title and abstracts were read on PubMed and selected on a yes, no, undecided basis. The studies that were marked yes and unsure were selected for full data extraction. All studies selected from the search were downloaded and inserted into an Excel Spreadsheet which was used as a record all of the studies (S1 Supplementary Information).

The second stage was a full paper extraction of studies selected in step one. For a study to be included in the review had to meet the following criteria: (1) be available in the English Language; (2) a study in a country where RVF is known to circulate; (3) provide quantitative or qualitative information on: serological information on RVF in animals and humans, qualitative description of wider social impacts, economic (economic data at local, regional, and national levels), epidemiological (morbidity and mortality data), social impacts (gender, sociocultural factors) to be extracted. The reviews were analysed to determine whether further information met the criteria above that could be extracted from them. The citations for the socioeconomic information were cross-checked to ensure no studies had been missed in the search.

**Fig 1:**
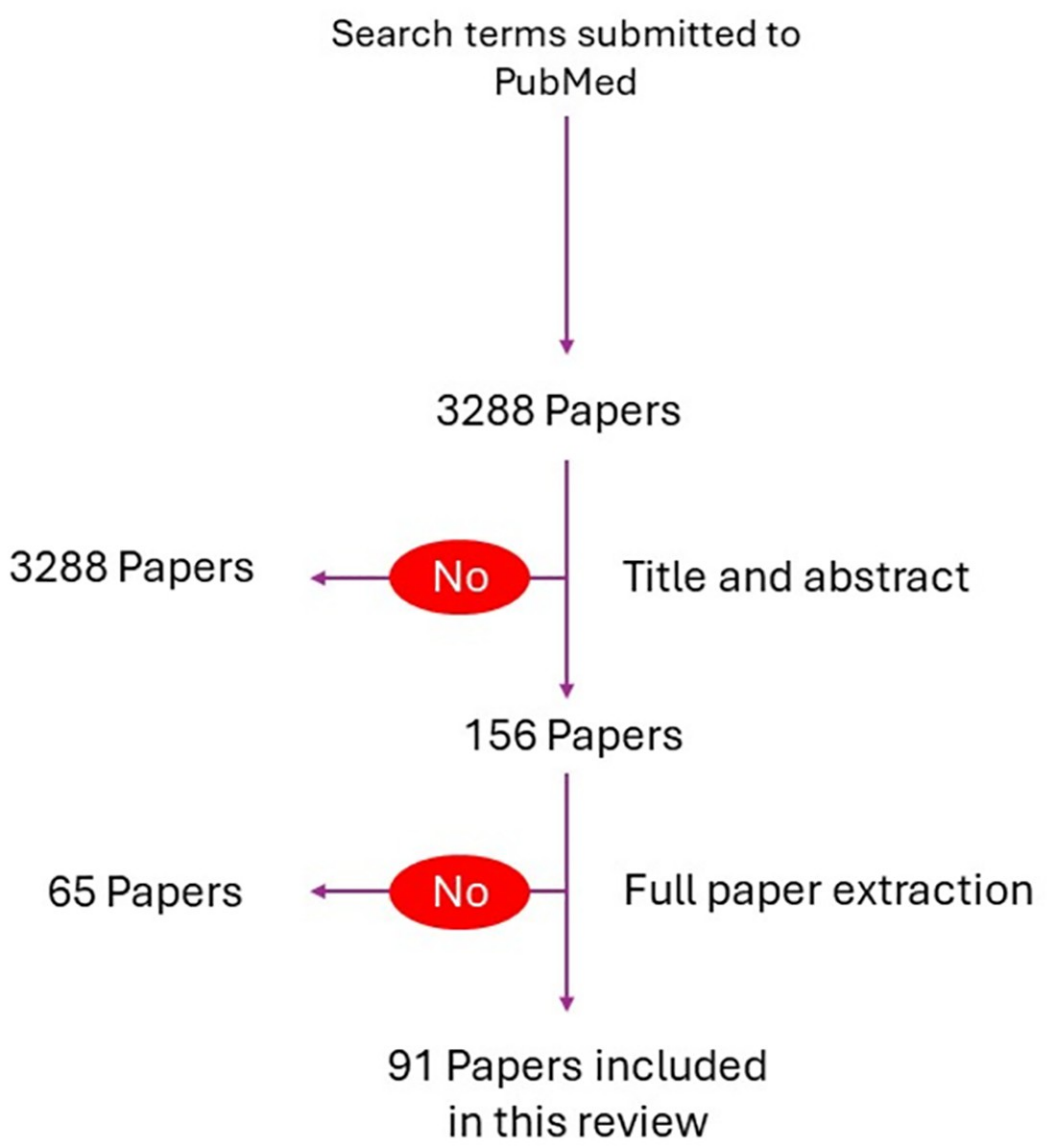
Illustrative flowchart of the data extraction.

The data was extracted and inserted to the Excel spreadsheet (S1 Supplementary Information). Data extraction categories included: Author, Year, Title, Source, Abstract, Country, Region, Level, Analysis, Functional Unit, and Comments (Fig 1).

## 3. Results

The initial search as described above returned 3,288 papers. The filtering of title and abstract excluded 3,132 papers. A total of 156 studies were included for full data extraction at the second stage. After further analysis, 65 studies were excluded (including duplicates) leaving 93 studies included in this review.

The 8 reviews found in the search were reviews conducted in specific endemic countries or regions (for example, East Africa or Senegal). The topics of the reviews included, lessons learnt from outbreaks, urbanisation, prevention, control, and licensed vaccines and new vaccines and therapeutics in development.

A total of 91 papers were included in this review and have been summarised in Table 1. The studies were categorised by country and type of study, which are not mutually exclusive. For example, mathematical modelling studies that modelled vaccination strategies would fall into 2 categories. Kenya was the country with more published studies (*n* = 35) which fit the inclusion criteria. This was followed by Tanzania (*n* = 9), South Africa (*n* = 8), Egypt (*n* = 6), Sudan (*n* = 5), Nigeria (*n* = 3), Mayotte (*n* = 3), Somalia (*n* = 2), Senegal (*n* = 2), and Madagascar (*n* = 2). Cameroon, Yemen, Mauritania, Tunisia, Rwanda, Saudi Arabia, Uganda, and Malawi each had 1 study. Studies included in this review (Table 1) were grouped into 3 main topics: health burden, economic impacts, and prevention and surveillance.

**Table 1:** Breakdown of the papers included in the rapid review. The green shading highlights the different types of study used for each country. There are 5 groups to show the number of studies: 0, 1–2, 3–4, 5–6, 7–8, 9–10; 0 is white and as the number of groups increases so does the green shading. The orange shading highlights the total number of papers included in this study for each country. The darker the orange colour the greater the number of studies. It must be noted not all methodologies are mutually exclusive, for example, some modelling studies modelled vaccination strategies.

Country	Mathematical models	Social studies	Value-chain analysis	Risk	Vaccination strategy	Economic evaluation	Epidemiology	Reviews	Total
Kenya	10	7	5	6	2	4	1	0	35
Tanzania	0	4	0	2	0	0	3	0	9
South Africa	0	3	0	2	0	1	2	0	8
Reviews	0	0	0	0	0	0	0	8	8
Egypt	4	2	0	0	0	0	0	0	6
Sudan	0	4	0	0	0	0	1	0	5
Nigeria	0	2	0	1	0	0	0	0	3
Mayotte	2	0	0	1	0	0	0	0	3
Somalia	0	0	0	0	0	1	0	1	2
Senegal	0	0	0	2	0	0	0	0	2
Madagascar	0	1	0	1	0	0	0	0	2
Cameroon	0	0	0	1	0	0	0	0	1
Yemen	0	1	0	0	0	0	0	0	1
Mauritania	0	0	0	0	0	0	1	0	1
Tunisia	0	0	0	1	0	0	0	0	1
Rwanda	0	1	0	0	0	0	0	0	1
Saudi Arabia	0	0	0	0	0	0	1	0	1
Uganda	0	1	0	0	0	0	0	0	1
Malawi	0	1	0	0	0	0	0	0	1
Total									91

### 3.1. Health burden

**Fig 2:**
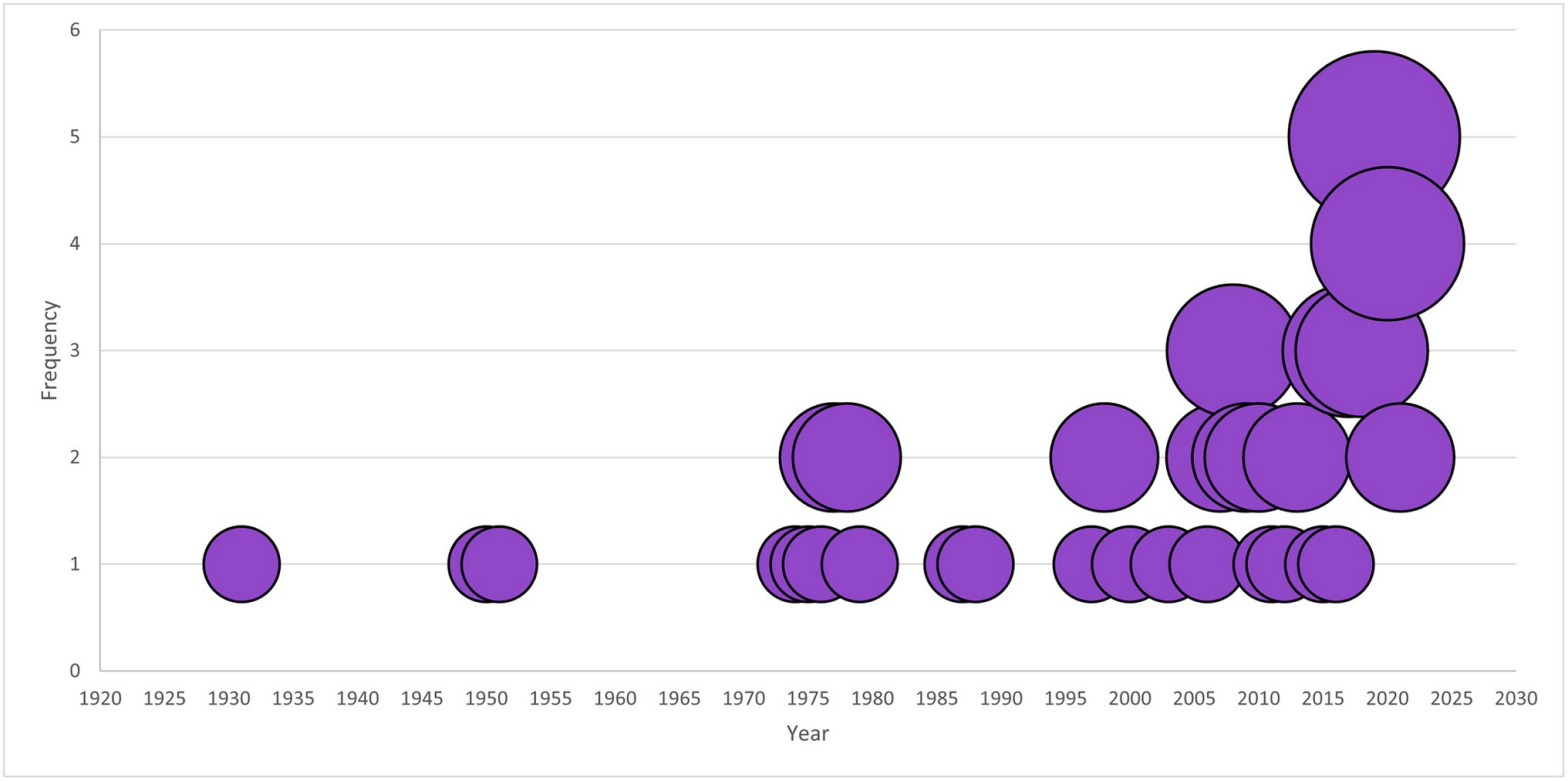
Illustrates all the years RVF incidence has been reported. The graph demonstrates RVF outbreaks are occurring at an increasing rate.

RVF outbreaks are becoming increasingly frequent, as can be seen in Fig 2. The history of RVF outbreaks, including morbidity and mortality in animals and humans can be seen in Table 2. RVFV was first identified in 1931, during an investigation into an epidemic among sheep on a farm in the Rift Valley in Kenya. Since then, major epidemics in Kenya have been reported in 1997 and 2006. Other major epidemics have been seen in Egypt (1977, 1993, 1994, 1997, and 2003), Tanzania (1997 and 2006), South Africa (1950, 1974, and 2010), Senegal (1993 and 1987), Somalia (1998 and 2007), Sudan (2000 and 2007), Saudi Arabia (2000), Mayotte (2008), and Mauritania (1987, 1993, 1998, 2003, 2010, and 2012).

**Table 2:** Morbidity and mortality in animals and humans during RVF epidemics. N/A, no available data in studies found in this review. Case fatality ratio is calculated by dividing the number of deaths caused by RVF by the number of RVF diagnosed cases.

Year	Countries	Morbidity and mortality						References
		Animals			Humans			
		Cases	Deaths	Case fatality ratio	Cases	Deaths	Case fatality ratio	
1931	Kenya	N/A	4,700	N/A	N/A	N/A	N/A	[17–20].
1950	South Africa	600,000	100,000	17%	N/A	N/A	N/A	[17,19–21]
1977	Egypt	N/A	N/A	N/A	200,000	569	0%	[10,17,22]
1978	Zimbabwe	70,000	10,000	14%				
1987	Senegal	1,715	N/A	N/A	273	16	6%	[17,23]
1988	Mauritania	N/A	N/A	N/A	N/A	224	N/A	[24]
1997	Kenya	89,000	478	1%	160,000	450	0%	[13,17–19]
	Somalia	N/A	N/A	N/A	28,000	170	1%	[17,25]
	Tanzania	N/A	N/A	N/A	89,000	478	1%	[17,19,26–29]
1998	Mauritania	343	N/A	N/A	90	1	1%	[24]
2000	Saudi Arabia	10,000	1,000	10%	883	245	28%	[13,19,30]
	Yemen	22,000	6,000	27%	1,328	166	13%	
2003	Egypt	N/A	N/A	N/A	45	17	38%	[10,17,22]
2006	Kenya	N/A	N/A	N/A	75,000* (684)	158	N/A	[13,17–19]
	Somalia	N/A	N/A	N/A	35,000* (114)	51	N/A	[17,25]
	Tanzania	32,000	4,200	13%	40,000* (264)	158	N/A	[17,19,26–29]
2007	Sudan	N/A	N/A	N/A	75,000* (698)	222	N/A	[13,17,19,26,31]
2008	Madagascar	N/A	N/A	N/A	10,000 (712)	26	N/A	
2010	South Africa	14,342	8,877	62%	242	26	11%	[17,19–21]
	Mauritania	N/A	N/A	N/A	N/A	N/A	N/A	[24]
2012	Mauritania	N/A	343	N/A	41	17	41%	[24]
2013	Mauritania Senegal	N/A 52	N/A 11	N/A 21%	N/A 35	N/A 0	N/A 0%	[32]
2015	Mauritania	291	3	1%	31	8	26%	
2016	Niger	156	156	100%	348	33	9.5%	[32]
2018	Kenya Uganda Uganda	130 N/A N/A	17 N/A N/A	13% N/A N/A	94 5(3) 4	11 N/A 2	12% N/A 50%	[33]
2019	Mayotte (France) Kenya Sudan	109 119 75	N/A N/A 12	N/A N/A 16%	129 21 293	N/A 11 11	N/A 52% 4%	[32]
	Libya	30	4	13%	0	0	0	
2020	Mauritania Sudan Kenya				N/A 1962 32(14)	3 79 11	N/A 4% 79%	[34]

The data of RVF burden in both animals and humans is erratic (Table 2) potentially due to the lack of active surveillance or published studies. English is not the first language in many endemic countries and this data may have been excluded by the search terms. Moreover, at the local level, veterinary departments will have their own records which may not be in the public domain. Outbreaks of RVF tend to follow the pattern of heavy persistent rain, generally in livestock first followed by human outbreaks. For example, in the major Kenya epidemic in 2006 it was estimated that there were 40,000 cases of RVF in humans but only a small proportion (264; 0.66%) was confirmed in the laboratory [17]. Moreover, there is less data available for livestock compared to humans and this review did not find any laboratory confirmed livestock RVF cases in any of the outbreaks reported in Table 2. This could be due to the lack of diagnostic capabilities in endemic countries and many rural locations could be inaccessible due to flooding.

**Fig 3:**
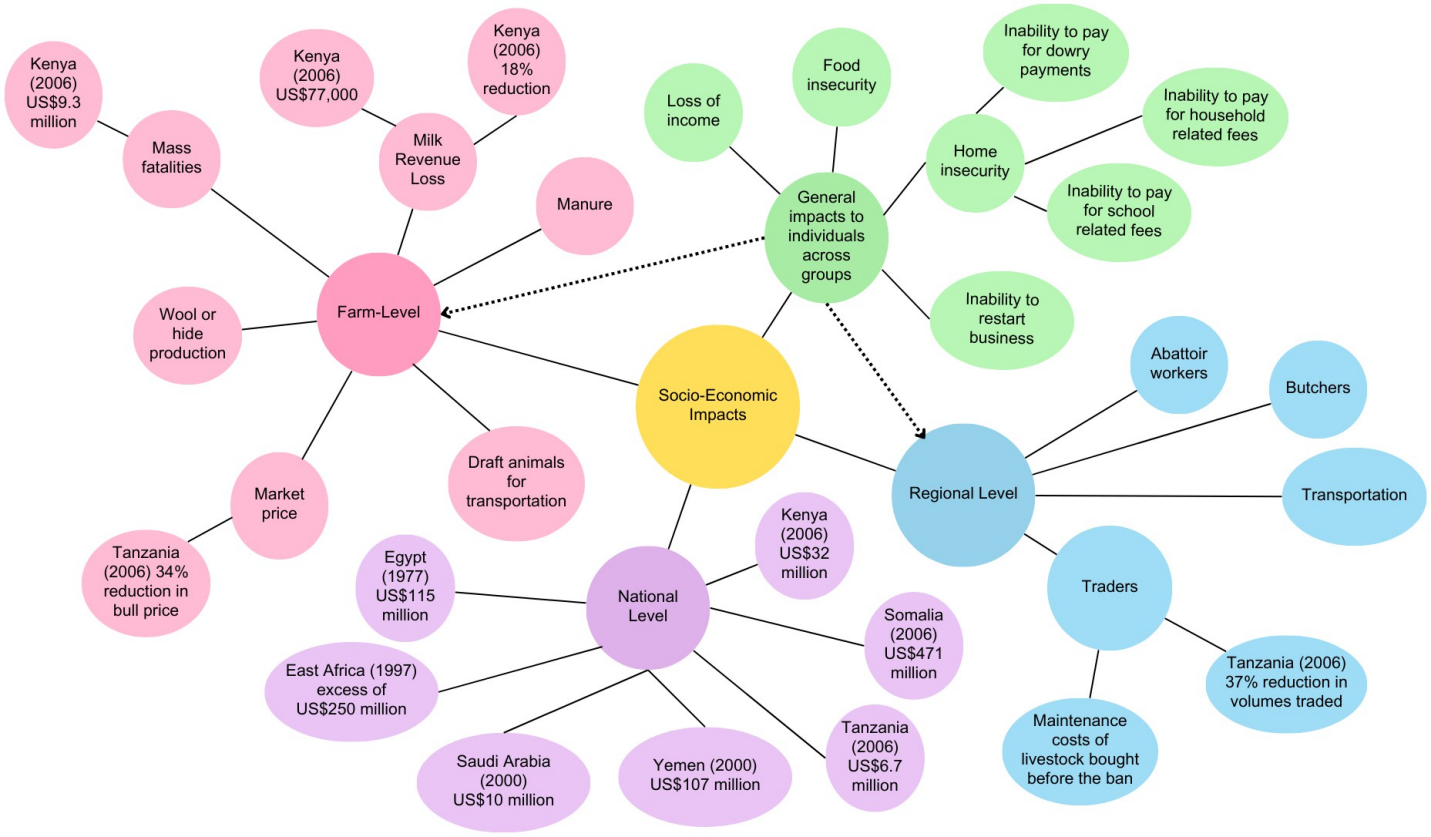
Illustrates the socioeconomic impacts of RVF outbreaks reported at farm, regional, and national levels. The socioeconomic impacts were extracted from the studies included in this review and then grouped into the following categories: general impacts (green) to individuals that would be experienced across all levels of society, farm-level (pink), regional level (blue), and national level (purple). The dotted lines indicate inter-connectivity between levels and the straight lines connect the level of society to examples of socioeconomic impacts at the different levels.

A summary of our results on the socioeconomic impacts of RVF outbreaks is available in Fig 3. Socioeconomic impacts are presented in terms of their reach; local, regional, and national. Local level is household impacts, regional is different impacts reported regions/districts within a country, and national level is impacts reported on the national scale. National level impacts are more frequently reported compared to other levels (household and regional) and are generally presented as a monetary figure in US dollars. The results show that there are general impacts that will be experienced across all levels. For example, food insecurity, loss of income, home financial insecurity, and inability to restart business [15,28,35]. In one study from Tanzania, pastoral participants stated that if there was an RVF outbreak, this would result in increased fatalities of their livestock would negatively affect their family, health, and finances [28]. Benefits of livestock stated by participants were centred around social impacts such as dowry payments, draft power, health, and ability to pay school and household-related fees [15].

Impacts experienced at farm level were mass fatalities of livestock (estimated to cost US$9.3 million in the 2006 Kenyan outbreak) and the inability to trade (locally and regionally) animal products, such as manure, milk, wool, or hides [15,35,36]. For those who could trade, it was reported reduced market prices significantly reduced their income [36]. These farm-level impacts had wider implications and are connected to the socioeconomic impacts at regional level. Again, there is a lack of data at the regional level, but it was reported a variety of industries have been impacted. For example, abattoir workers, butchers, transportation, tourism, chemical, petroleum, public health, and import/export industries [15,28,35,36].

The evidence presented here demonstrates the interconnectedness of the socioeconomic impacts of RVF and that all levels of society are impacted. However, more needs to be done to gain a greater understanding of how different industries are impacted along the value chain and how this impacts the wider society. For example, no studies found in this review discuss the longer-term impacts of mass fatalities in livestock on the herd or the farmers. No studies evaluated the mental health of health workers during outbreaks when healthcare services were stretched. Lastly, only 3 studies discussed women in the context of RVFV, 2 discussed the gendered barriers to vaccine uptake, and the other demonstrated increased risk of abortion in RVFV–positive women.

### 3.2. Occupational risk

The evidence found in this review suggests that most outbreaks occur within livestock first before zoonotic transmission events to humans. Therefore, individuals who work within the livestock production system are at an increased risk of RVF exposure. For example, pastoral communities and abattoir workers.

Pastoral communities are seen as having the greatest risk to RVF exposure. Pastoral communities were the most frequently sampled in the studies included in this review, with other production systems rarely being stated. It is thought pastoral communities are at greatest risk of RVFV exposure because of time spent with their livestock [4,24,32,35–39]. Pastoral communities are subsistence farmers who rely on their livestock for survival, sustenance, and income [15]. Male community members can travel large distances with their livestock in search for pasture and water. However, other industries are at risk of RVF exposure (e.g., abattoir workers and veterinarians [4,17,37–40]). For example, during 2008 to 2011 in South Africa, there were 254 confirmed cases of RVF, 60% were livestock farmers; 13% animal health workers; 11% abattoir worker, butcher, or hunter; 2% farm resident (non-worker); and 15% were nonanimal-related occupations [40].

Various studies have sought to understand the Knowledge, Attitudes, and Practices (KAPs) among livestock farmers across various endemic countries (Kenya, Tanzania, Sudan, and South Africa) [15,28,41–46]. Multiple KAPs have been conducted in Nigeria, where RVFV is known to circulate but is considered non-endemic [47–49]. The majority of studies suggest low levels of knowledge regarding RVF (Kenya, Nigeria, Sudan, and Tanzania) with some participants showing higher knowledge of zoonoses (South Africa). Alhaji and their team found sociodemographic characteristics of pastoralists influenced their knowledge of RVF in Nigeria [47]. Therefore, KAP studies have shown an increased level of knowledge does not always lead to good practices.

Behaviours that increase risk of RVFV exposure were also reported. These behaviours are and not limited to slaughtering animals at home for human consumption [41], the consumption of animal products (e.g., unpasteurised milk, blood, and meat), and the use of ghee and fats to treat common infections such as diarrhoea and ulcers [35,50]. Limited knowledge combined with at risk behaviours increase the risk of RVFV exposure. This is because if the animal slaughtered or animal products consumed were from a sick animal this increases the risk of RVF infection.

#### 3.2.1. Gender

Most epidemiological studies found in this review (10/17, 65%) contained a majority of male participants (range 57 to 93 (% of male), *n* = 4,471) [24,37,39,41–47,49–55]. Six studies had a minor female majority (range 50.1 to 58.5 (% of females) [43–45,50,54,56]. One study had strong female selection with 74% female participants [47]. No study specifically investigated the occupational risks of women and RVF.

Three studies in different countries found males were 3 times more likely to be seropositive than women [17,24,31]. It has been suggested that the increased time spent in close contact with their livestock, males of increasing age have a greater chance to be exposed to RVFV [50]. Women’s main responsibilities are to manage milking duties, rearing the young livestock, and the sales from dairy products [43].

Vertical transmission in women has been documented twice: Saudia Arabia (2000) [57] and Sudan (2011) [7]. In Saudi Arabia, a five-day-old infant was administered to hospital with respiratory issues and died 2 days later [57]. It was later found that 4 days prior to the delivery, the mother had developed RVF-like symptoms and had potentially been exposed by being in contact with sick or aborting animals during the RVF outbreak. In Sudan, a study conducted in 2011 of 130 pregnant women found 28 (18%) of women were positive for RVF infection. Of these 28 women, 54% had a miscarriage compared to 12% of the women who were RVF negative [7]. Patients positive for RVF also had higher rates of bleeding, joint pain, and malaise [7]. The same Sundanese study [7] reported vertical transmission in women.

#### 3.2.2. Sociocultural practices

Religious festivals have been shown to be amplifiers of RVF transmission as seen by the introduction of RVF into Saudi Arabia in 2000 [58]. The resulting outbreak inflicted severe disease and economic losses for the first time outside of the African continent [29] and is thought to have originated through livestock trade across the Red Sea [17,20]. This is supported through phylogenetic analysis as the strain responsible had close relationships to the Kenyan 1997 outbreak [59]. Moreover, the outbreak coincided with the religious festival Eid and the increased number in livestock at the end of rainy season may have provided a suitable environment for the amplification of RVF. The outbreak resulted in 1,328 cases and 166 deaths in humans [13,17,19,26] and 22,000 reported cases and 6,000 deaths in animals [13,17,59].

There are many factors that might result in increased risk of RVF transmission at religious festivals. Large influxes of livestock into countries in order to meet the increased demand of livestock and animal products can result in increased RVF transmission along the migration route [60]. Multiple studies have demonstrated that if a religious festival coincides with mosquito season, this significantly increases the risk of RVF outbreaks [22,60,61]. Livestock sacrifice at religious events have been shown to be important transmission routes of RVF [58]. Rituals take place in large groups and, if infected animals are slaughtered, this increases the risk of RVFV exposure via animal blood and aerosols from infected livestock during slaughter. During the 2006 outbreak bans on sacrifice at religious events and weddings were supported by local imams and sheikhs (religious leaders). This proved a critical factor in reducing the mortality and morbidity of animals and humans alongside the government-led restrictions [62]. This suggests policy development at the local level can be effective in the control of RVF when planned alongside local leaders.

### 3.3. Economic impacts

Data on the economic impact of RVF outbreaks are limited. Outbreaks costs have been estimated in Kenya, Tanzania, and South Africa. The majority of the economic data is concentrated on the direct losses experienced at the farm and national levels. However, there are many more industries along the production chain that would be affected by an RVF outbreak. The financial impacts of RVF outbreaks on countries have been summarised in Table 3. The wide variety of economic impacts at the farm, regional, and national levels have been illustrated in Fig 3.

**Table 3:** A summary of the economic impacts across the farm, regional, and national levels. N/A, no available data found in this review.

Year	Country	Estimated livestock abortions (%)	Estimated farm-level economic impact (US$)	Estimated regional-level economic impact (US$)	Estimated national economic impact (US$)	References
1977	Egypt	Sheep = 80–100	N/A	N/A	115,000,000	[13,20]
1997	Kenya	N/A	N/A	N/A	In excess of 250,000,000	[13]
	Somalia	N/A	N/A	N/A		
	Tanzania	N/A	N/A	N/A		
2000	Saudi Arabia	Goats = 90 Sheep = 90	N/A	N/A	10,000,000	[20]
	Yemen	N/A	N/A	N/A	107,000,000	[13]
2006	Kenya	Cattle = 47 Goats = 63 Sheep = 70	Producers = 9,300,000 Milk production = 77,000	Interviewed participants Traders Unsold livestock = 1,300 Slaughterhouses = 1,000 Butchers = 900	66,000,000	[35,63]
	Somalia	N/A	N/A	N/A	471,000,000	
	Tanzania	Cattle = 8 Goats = 31 Sheep = 13	N/A	N/A	6,700,000	

#### 3.3.1. Farm level—East Africa

The economic impacts found in this review were reported for the 2006 East African (Kenya, Somalia, and Tanzania) outbreak. The main impacts on producers were caused by the loss of animals, which in turn had impacts on food access and availability and future income. During the same outbreak, high abortion rates in cattle, sheep, and goats were observed [63]. Similarly, in Saudi Arabia outbreak in 2000, it was estimated 90% of goats and sheep experienced spontaneous foetal abortions [20].

An important study identified in this review was a value chain analysis conducted in Kenya [35]. The direct losses to producers caused by the mass fatalities in livestock were estimated at approximately US$9.3 million dollars [35,64]. They also estimated the milk revenue lost to be in excess of US$77,000 [35]. This was the only study in which the authors calculated indirect losses to farmers. It has been estimated there was an 18% reduction in milk production in 2019 when compared to 2015–2018 [65], which cost US$207,283. Other examples would be loss of manure, draught animals for transportation, wool or hide production, and revenue lost in days out of work. A study conducted in Tanzania estimated the price of a mature bull dropped from US$238 to US$158 during the 2006 Tanzanian outbreak [27].

#### 3.3.2. Regional level

During the East African (Kenya, Somalia, and Tanzania) outbreak in 2006, it was reported there was a 37% reduction in the volumes traded in Tanzania [66]. It was reported in Kenya many butchers and traders could not restart once the ban was lifted due to the lack of financial capital [35]. In addition, the demand for red meat shifted to other meat products, leading to shortages and price rises in other markets, e.g., chicken, pork, and vegetables [15].

#### 3.3.3. National-level impacts

**Fig 4:**
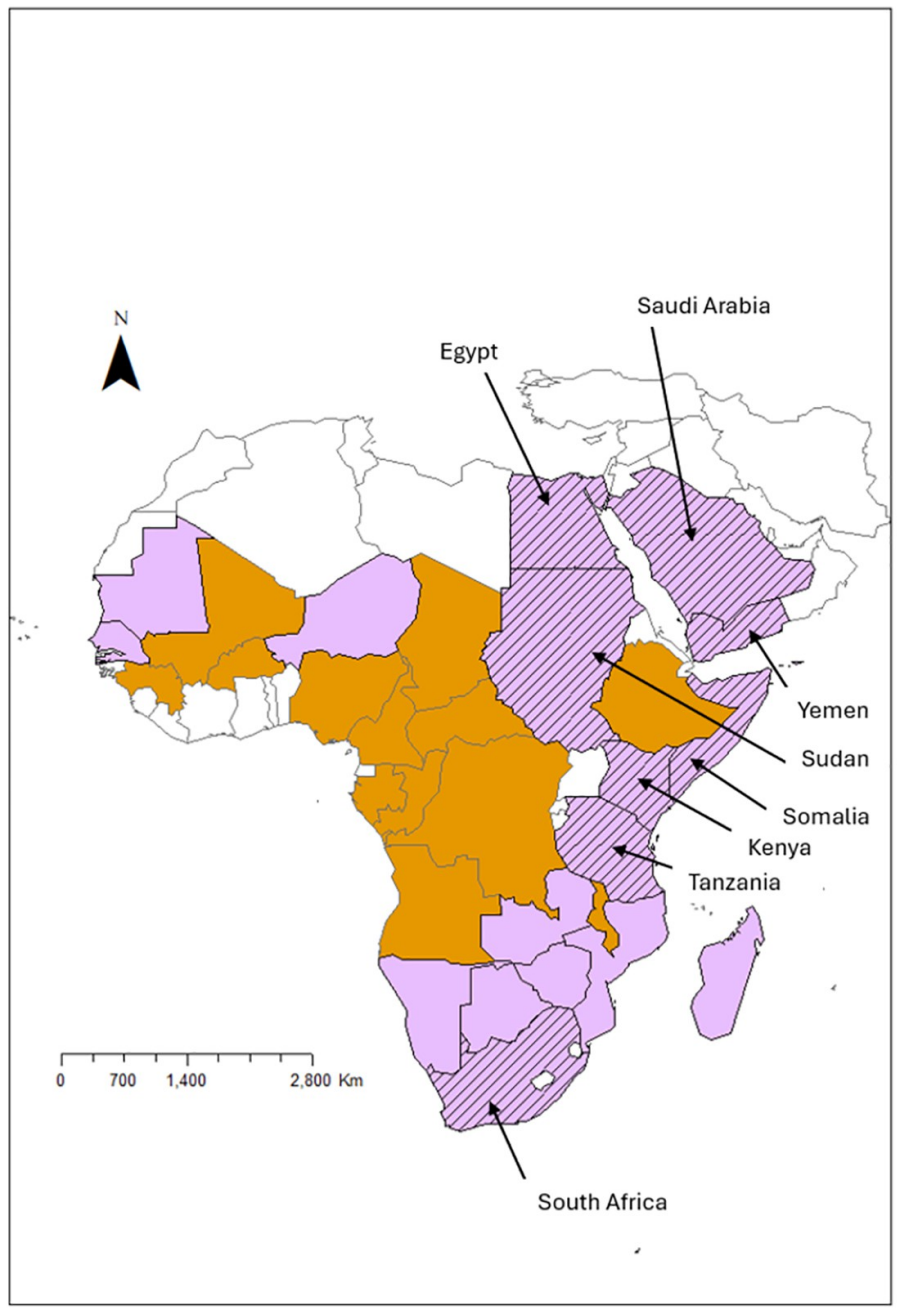
Is an illustrative map of Africa and Southwest Asia. The purple countries report endemic disease, orange countries report few cases and sporadic outbreaks, and grey countries the status of RVF is unknown. The dashed lines highlight the countries where economic impacts have been found in this review. The endemicity data is based on the data from epidemiological update and risk of introduction to Europe [74]. The shapefiles to create the map were downloaded from DIVA-GIS website (https://diva-gis.org/). Map was created using tools provided in ArcGIS 10.8.1. (Esri Inc, 2016).

Fig 4 illustrates the range of economic impacts at the national level found in this review. This review found national-level economic data of 7 countries (Egypt, Kenya, Tanzania, Saudi Arabia, Somalia, South Africa, and Yemen) [4,13,17,19–21,26–29,59,64,67–72] for previous outbreaks (1976 to 2007). Published papers from Sudan did not provide an estimation of economic impact but case numbers for the 2007 outbreak have been estimated [26,71,72]. Out of the data available, Somalia has been severely impacted by RVF outbreaks. This can be seen from the outbreaks in 1998 to 2003 and 2007 were estimated to of cost US$330 and US$471 million dollars [13,19,29]. It is difficult to draw comparisons between estimations for different countries; currency value varies over time, each country’s livestock population differs in size and type of animals included, while methods of estimation are likely to be different between studies.

In 2000, there was a geographical expansion of RVFV across the Arabian Peninsula into Yemen and Saudi Arabia [13,17,19,26,59]. The outbreak in 2000 was estimated to have cost Yemen US$107 [19]. Yemen was the only country where an economic estimation was calculated for impacted industries: US$15 million to the livestock industry, US$30 to tourism, US$50 million to exportation, US$12 million to public health, and US$0.1 million on vector control [13]. The economic impact in Saudi Arabia was estimated to have cost in a range of US$75 to 90 million [71]. In an attempt to bring the outbreak under control, Saudi Arabia banned the importation of livestock from the horn of Africa. This ban had severe economic impacts on Somalia as discussed above.

The East African outbreak in 2006/2007 severely impacted Somalia as described above followed by Kenya (US$32 million) and Tanzania (US$6 million) [4,13,27,28]. The impacts to their GDP were 5% in Somalia, 0.1% in Kenya, and 2% in Tanzania. Kenya was the only country where the public health burden was described. The total burden in disability-adjusted life years (DALYs) was estimated to be approximately 4,000, with an estimated 3.4 DALYs per 1,000 people, and the household costs to be US$120 for every human case reported [69]. DALYs are the accumulation of life lost and years lived with a disability [73]. Moreover, hospitals incurred extra costs of US$70.8 per patient for the diagnosis, treatment, and protective equipment [68], resulting in a severe burden on the public health system. A study in Tanzania found patients on average remained in hospital for 120 days before discharge. Also, of the 309 laboratory-confirmed human cases of which 144 died, a case fatality ratio of 46.6% [70]. The combination of this would have also put severe strain on the public health services in Tanzania. Phylogenetic analysis of the 2007 outbreak in Sudan revealed close ancestry to the 2006 outbreak in Kenya. Further supporting the transboundary nature of RVFV. No economic or livestock data for the outbreak in Sudan was found in this review. However, it was reported the livestock export industry decreased significantly [31].

Although, the economic data is patchy, the evidence available demonstrates the severe economic impacts that have occurred in endemic countries and countries who experienced RVF outbreaks for the first time. RVF outbreaks are not isolated to one industry but impact a wide variety of industries along the value chain. For example, from the data available the livestock industry and consequently the export industry were the most severely impacted industries reported in the 8 countries above. Other industries reported to be impacted due to RVF outbreaks were public health, tourism, chemicals, petroleum, trade, transportation, and vector control. There are inter-relationships between outbreaks and trade is a major contributor to RVF dissemination between countries. This highlights the transboundary nature of RVFV, and Fig 4 illustrates this through the close proximity between many of these countries.

### 3.4. Prevention and surveillance

There are no licensed human vaccines and only a handful of licensed veterinary vaccines used around the world in animals. Other preventative measures used have been quarantines, bans on the sale and movement of livestock and international trade bans. Table 4 summarises different prevention and surveillance policies that have been recently implemented for the control of RVF.

**Table 4:** A summary of the different prevention and surveillance techniques within different endemic countries.

Country	Prevention and surveillance	Comments	Citation
South Africa	Vaccination programmes Vaccines used: Smithburn Clone 13	Since 2010, 19 million clone 13 vaccines administered. A study in South Africa found 26% of farmers did not vaccinate their livestock	[29,40]
Kenya	Development of One Health strategic plan for the prevention and control of zoonotic diseases in Kenya Livestock are quarantined at abattoirs for 2–6 days Reducing the risk to abattoir workers Market inspections Vaccination programmes Vaccine used: Smithburn Control measures of previous outbreaks Movement restrictions Ban on trade Quarantine Warnings on the consumption of raw milk, uninspected meat, and slaughtering of animals	The development of the plan has seen the efficient containment of an outbreak in 2018.	[62,63,75–77]
Egypt	Imported livestock: Quarantine Vaccination	The vaccination coverage varies greatly between governates. For example, 30% of sheep and 60% of cattle have been vaccinated.	[78]
Somalia	Market inspections Quarantine	The study found market inspection and quarantine had little impact on the detection of RVF and the cost-effectiveness was similar compared to quarantine alone.	[79]
Senegal	Development of map of migration routes between markets and abattoirs		[80]

#### 3.4.1. Barriers to vaccine uptake

*3*.*4*.*1*.*1 Gender*. A study investigated the gender barriers to livestock vaccine uptake in Kenya and Uganda. The study took place in the Kenyan counties of Murang’a and Kwale and the Ugandan districts of Arua and Ibanda. A total of 645 (323 females and 322 males) individuals took part in the study, with 317 from Kenya [43]. The participants identified 37 unique barriers to vaccine uptake. Men reported the main barrier of vaccine uptake was cost, while women identified the main barrier to vaccine uptake was men’s unavailability. Both men and women identified location of vaccine centres as the main barrier for women.

A stakeholder analysis in Rwanda found that many vaccines including those for RVF are inaccessible for many women small-scale farmers [81]. The barriers identified by different stakeholders for women entering the livestock vaccine chain were grouped into 4 categories: laws and regulations; access to resources including credit, vaccines, and infrastructure; cultural norms and gender stereotypes limiting women’s participation in the value chain; and weaknesses with vaccine distribution and training opportunities. Larger systemic engagement of all stakeholders and recognition of women’s roles in the livestock community is required in order for women to have greater access to the vaccine value chain [48,51,52].

*3*.*4*.*1*.*2 Willingness to pay*. Three willingness to pay studies have shown cost to be a major barrier to vaccine uptake; 2 were conducted in Kenya [43,54] and 1 in South Africa [55].

The first (Mutua) study implemented 3 different pricing structures across 4 study regions in Kenya and Uganda [43]. The locations were purposefully selected due to recent outbreaks and vaccination history and focus groups were used to collect the data. In Kwale in Kenya the vaccine was fully subsidized, but farmers were charged a small fee per head of cattle [54]. This was seen as a deterrent to some farmers, especially those that owned large herds. Moreover, farmers incurred extra costs to transport their herds to the vaccination centres. Similar cost barriers were seen in Tanzania due to extra veterinarian costs. For example, for the producers to receive treatment from veterinarians they had to pay for the costs of fuel in addition to the treatment. This is unattainable for many producers who already live in low-resource settings. In contrast, a willingness to pay study carried out in the same regions in Kenya found the average willingness to pay to be 40% higher than the estimated cost of the vaccine [54], a demonstration of willingness to pay for vaccines.

In the Mutua and Wanyoike studies all participants were livestock producers and farmers [43,54]. The participants in the Wanyoike study reported higher levels of wealth and greater knowledge of RVF [54]. Although this was not included in the Mutua study [43], the increased wealth and prior knowledge of RVF in participants of the Wanyoike study [54] could explain why they were willing to pay more for a RVF vaccine.

The third study in South Africa found 26% of participants did not vaccinate their livestock. Similar to the Kenyan and Tanzanian willingness to pay studies the major barriers to vaccine uptake were cost, vaccine complacency and past experience [55]. The vaccine is sold in vials containing 100 doses and for many smallholders this exacerbates the problem as they have fewer animals. By the time booster vaccinations are required for their herds, the vaccine will be ineffective due to the cold chain requirements. However, *s*ome farmers grouped together to overcome the financial barriers [55].

To compare the price of the vaccines, we converted the local currencies into US dollars, using the 2023 exchange rate. In the South African study, the farmers could buy vials with 50 doses of the vaccine at US$0.21 per dose. The Kenyan studies vaccine prices ranged from US$0.36 to 0.90 and in Uganda the vaccine prices ranged from US$0.54 to 0.80 per dose. Overall, the South African vaccine is much cheaper compared to Kenya and Uganda.

## 4. Discussion

### 4.1. Economic impact

There is a lack of economic data at national, regional, and local levels of society. From the data that is available (Table 3), this review reports economic data for 8 countries (Egypt, Kenya, Saudi Arabia, Somalia, South Africa, Sudan, Tanzania, and Yemen). This review did not find economic data for 10 (out of 18) countries considered endemic at any level. Although the economic data is lacking the comparison of livestock mortality data in Table 2, it is evident RVF outbreaks can significantly impact national economies.

Trade is an important risk factor in the introduction of RVF into new susceptible environments. At the local level, pastoralists can travel across borders between countries to sell their livestock at markets. The different herds can be in close contact providing an opportunity for RVFV transmission. The Illegal trade of livestock is another pathway in which RVF can be introduced into susceptible populations. It is thought illegally traded livestock may have been the pathway which introduced RVF into Mayotte prior to the 2018 epidemic. The porous borders and the lack of checks and documentation for this livestock could increase the risk of exposure of RVF to humans and livestock in the country they have been traded. Illegally traded livestock will be cheaper than market inspected meat. Individuals who buy these livestock or animal products tend to be of lower socioeconomic status and could increase their risk to exposure of RVF. The ability to trace the origins of the livestock is critical in an outbreak because this will aide in the control and spread of RVF.

At the international level, trade without strict checks is also seen as an important risk factor of RVF transmission. A prime example is it is thought the importation of livestock resulted in the introduction of RVF into Saudi Arabia in 2000. As a result, Saudi Arabia banned the importation of livestock from the horn of Africa [12,15,29]. At this time, Somalia was the second largest country (22.89%) in which Saudi Arabia imported live animals from. Moreover, Somalia exported 92.17% of their live animals to Saudi Arabia [82]. The loss of nearly a quarter of Saudi Arabia’s import could have led to food shortages and price rises for other animal products, similar events were reported in Tanzania [15]. The trade embargo would have had devastating impacts to the Somalian economy because of the heavy reliance on exports to Saudi Arabia.

International trade of livestock is heavily relied upon in Africa and Asia. For most recent data available, it is estimated 92.5% of livestock imports from Africa are traded within Africa and Asia (data from 1995 to 2021) [82]. The biggest importers of African livestock are Saudi Arabia (28.53%), Oman (18.07%), South Africa (14.74%), and Egypt (12.05%) [82]. Therefore, trade bans to limit the spread of an RVF outbreak could severely impact countries at all levels of society.

This data highlights the transboundary nature of RVF and how trade local and international can increase the risk of RVF outbreaks. This review found little economic data for industries along the production chain apart from one study conducted in Kenya [35]. However, from the data collected on trade embargoes, quarantine, livestock movement restrictions will have had significant economic impacts along the value chain. Greater implementation by both importers and exporters of policy designed for the prevention and transmission of RVF could aid in reducing the economic impacts for future outbreaks.

### 4.2. Sociocultural factors

Gender is a fundamental aspect of social research that must be considered for RVF. This review has found women tend to have varying roles within pastoral communities, suggesting varying rates of risk depending on the activities they carry out. The risks of RVFV exposure for women are relatively unknown. A prime example of this is vertical transmission in women as shown in the Saudi Arabian and Sudanese studies [7,57].

No study included in this review specifically investigated the impacts for women. In this review, 11 (out of 17) studies had a male bias with an average of 67% male participants. Six seroprevalence studies were included in this review; 3 did not provide sex aggregation of occupation, 2 studies found no statistically significant differences between male and females, 1 found males were more likely to be seropositive. Although a small sample size, it raises the question why studies bias towards the male gender if there is no significance between seropositivity levels between men and women. The same can be seen in KAP studies where no studies provided data to understand the difference in KAPs for male and female participants. One study stated men had significantly better knowledge and 1 reported no difference in knowledge between men and women.

In future studies, it would be useful to understand the occupation of women and their roles within communities instead of being grouped together under “housewife.” This would enable the evaluation women’s risk to RVF. Urgent research is required to fill this knowledge gap and future policy should be designed with consideration of risks to both males and females.

Sociocultural beliefs within pastoral communities can be barriers to vaccine uptake and agricultural extension services. For example, in some countries women are not able to travel to vaccination centres and interact with the medical staff of the opposite sex. Therefore, they are required to rely on male friends and family to assist them in vaccinating their livestock and resulting in less access to agricultural extension services. This is supported by the gendered barriers to vaccine uptake study and vaccine chain analysis in Rwanda [43,81]. Empowering female farmers can increase vaccine uptake and reduce the number of livestock lost to infectious disease [83]. It has been shown that women dedicate in excess of 90% of their income on to meet household needs such as improving health and nutrition [84]. This could be because women earn less than men and so a higher proportion of their income is spent on basic needs. Not only are empowering women an effective way to combat household poverty and food insecurity, but it is also a method of achieving sustainability goals. To overcome gender as a barrier, it is critical to include more females in stakeholder discussions.

This rapid review found that the majority of studies were conducted on livestock with little consideration given to sociocultural aspects. The review highlights a variety of barriers to vaccine uptake in pastoral communities. For example, cost, lack of willingness, lack of access to vaccines, and lack of funds along with other social aspects. Many countries now implement prevention and surveillance methods for the control of Rift Valley fever (Table 4) but there is evidence to suggest vaccine uptake within countries is low.

### 4.3. Prevention and surveillance

Endemic countries have implemented policies for the control of RVF (Table 4), such as quarantine and vaccination of imports and exports, but their effectiveness has not been assessed. For example, in South Africa 26% of smallholder farmers did not vaccinate their livestock. In Egypt, it is reported RVF vaccine uptake in livestock was found to be as low as 20% to 30% in some governates, with lower coverage in other livestock [78]. Therefore, evaluation of why there is low vaccine uptake within farming communities is required. This would enable the development of more efficient policy that could increase vaccine uptake and aide in the control of RVF.

Low levels of compliance suggest that the policies implemented for the control of RVF in times of outbreak are designed without the consideration of farmers’ needs and motivations. For example, during the 2006 East African outbreak (Kenya, Somalia, and Tanzania), a ban on the consumption of raw milk, livestock movement, and animal slaughter was implemented, which is in line with the Food and Agricultural Organisation (FAO) recommendations [33]. However, in Tanzania participants reported the consumption of animal products and the movement of livestock in search for open markets took place [15]. The continued movement of livestock could have led to further outbreaks outside of the quarantine area. For example, in Kenya the outbreak in Baringo occurred 6 weeks after the outbreak in Garissa [85].

There are multiple factors that could contribute to the lack of compliance. For example, lack of trust towards the government or health professionals. KAP studies demonstrated low levels of knowledge of RVF in pastoral communities. Therefore, pastoral communities may not recognise the link between transmission pathways of RVF and bans implemented by the government. In order to have greater local compliance continued collaboration of livestock farmers, public health and veterinary services are required to improve communication to increase compliance and reduce transmission of future outbreaks. This collaboration will help build trust in these rural communities which in the future could lead to better utilisation of livestock farmers in surveillance and help improve vaccine uptake. A prime example of effective collaboration between public health officials and veterinary health can be seen in the joint human and animal vaccination programmes (JHAVP) in Chad [83,84,86–89]. The effective collaboration helped rebuild trust with rural communities who felt neglected, resulting in higher vaccine uptake in animals (anthrax, blackleg, pasteurellosis, and contagious bovine pleuropneumonia), women (tetanus), and children (diphtheria, whooping cough, tetanus, and polio).

Multiple studies have demonstrated the importance of increased surveillance and the need for the integration of public health and veterinary services. This is because the majority of RVF positive cases in humans can be linked to close contact with livestock.

During an outbreak, greater emphasis of symptoms in public education campaigns could lead to a greater number of individuals reporting. For example, in Mayotte, during the last epidemic it was estimated only 1.2% of human cases were reported [15,65,90–92]. Symptoms in humans are often nonspecific, which may result in individuals being unaware they are infected with RVFV. In participatory studies, many participants had not received a formal education and could not read. Therefore, posters would be an ineffective way to share important information regarding RVFV transmission.

As discussed above, trade is an important risk factor in RVFV transmission and pastoral communities can travel large distances to trade. Kenya and Senegal have begun developing maps of migration routes taken by livestock to gain a greater understanding of RVFV transmission along these routes. For example, through the creation of a map of livestock movements in Western Kenya (by stakeholders from 2 slaughterhouses), they traced seropositive livestock back to their original market in Migori county [77,93]. Through greater data collection and mapping of livestock movements high-risk areas can be identified and more efficient surveillance can be designed within these areas at times of high risk.

Predictive weather technology is improving and has been seen to predict weather events which increase the risk of RVF outbreaks 3 months in advance, e.g., 2006 East African outbreak. However, the current production time for vaccines is 4 months. One way to reduce the socioeconomic burden of disease outbreak could be to set up vaccine banks and to mobilise teams to the hotspots before such weather events occur, ensuring the infrastructure is set up in time for the vaccine’s arrival.

Greater data collection during outbreaks in both public health and veterinary fields can enable a better understanding of transmission dynamics and evaluation of RVF risks for livestock and humans. This would also enable greater collaboration with subsistence farmers, leading to better designed policy for points of intervention, vaccination, and other control measures.

Greater data collection can also enable more accurate mathematical models of RVF. Mathematical models can be used in policy development to assess the impact of potential control strategies on RVF. Modelling vaccination in Kenya has shown a variety of livestock vaccination strategies are effective at controlling RVF outbreaks [94]. Interestingly, modelling suggested that a hypothetical human vaccination strategy (vaccinating 80% of individuals from in the farming group) in Mayotte would not result in a significant reduction in the number of human cases as compared to the livestock vaccination strategies [95]. Vaccinating at risk occupations might be an effective vaccination strategy to reduce the number of human RVF cases. Future research could include economic evaluation of different vaccination strategies. This would have enabled the DALYs of each strategy to be estimated.

## 5. Conclusions

The evidence of this rapid review suggests that: (i) individuals of a lower socioeconomic status are at disproportionately higher risk of RVF infection; (ii) greater knowledge of RVF does not always translate into better practices by farmers; and (iii) there is a gendered knowledge gap of risks of RVF exposure for women.

No studies have fully explored the wider societal impact of RVF outbreaks; for example, the long-term impacts of RVF outbreaks on pastoral communities have not been assessed. Disaggregated data collection in both animals and humans will enable evaluation of these impacts. Furthermore, the evidence suggests low compliance with policy within low-resource settings. Further work is required to collaborate with local communities on the roles of specific socioeconomic risk factors, which in turn can aid in the development of relevant local control measures.

Operationalising One Health can help achieve effective policy on prevention and control of RVF at the animal–human interface. Through a better understanding of the socioeconomic impacts of RVF and incorporation of gender and other wider societal factors could lead to collaboration and compliance of the local communities will lead to improved policies for the prevention and control of RVF.

## Supporting information

S1 Supplementary Information This Excel document contains all studies included in this review. (XLSX)
